# Toward Affective Interactions: E-Motions and Embodied Artificial Cognitive Systems

**DOI:** 10.3389/fpsyg.2022.768416

**Published:** 2022-04-13

**Authors:** Alfonsina Scarinzi, Lola Cañamero

**Affiliations:** ^1^CY AS Institute for Advanced Studies, CY Cergy Paris Université, Cergy, France; ^2^Zentrale Einrichtung fuer Sprachen und Schluesselqualifikationen (ZESS), University of Göttingen, Göttingen, Germany; ^3^ETIS Lab, CY Cergy Paris Université, Cergy, France

**Keywords:** embodiment/bodily experience, emotions and affectivity, human-robot-interaction, e-motion and artificial agents, affective interactions between humans and robots, movement and emotions

## Introduction

Emotion is at the core of the human experience. It enriches and makes meaningful and memorable our lives, our practices, our places, our tools, locations in a landscape, ritual actions, habits, and interactions with each other and with the environment. It represents bodily mediated evaluations (Petrides, 2018). In artificial agents like autonomous robots, emotions can be considered to be an integral part of a behavior-based robot's architecture. As “second-order” control mechanisms for fast adaptation to changing circumstances, they can alter motivational priorities and behavior execution and improve action selection, which is relevant for the robot survival, as Cañamero and Gaussier (2005) and Cañamero (2019) remark.

It is acknowledged that emotion and affectivity—the ability to move to emotion—are embodied, situated, and distributed in the environment, related and subordinated to the movement (Fuchs and Koch, 2014). Any interaction that is colored with emotion is affective. Emotions can be considered to be affective responses to events of concern to a sense-maker or agent, implying bodily changes and motivating specific movements and behavior and are articulated spatially through the making of spaces and environments that actively engage the sense-maker or agent in the regulation and control of movement (Tarlow, 2012; Kaczmarczyk, 2013). The properties of a garden, for example, can provide joyful visual, acoustic, olfactory, and tactile properties that can influence the way the sense-maker walks to explore it. A garden can be full of affective affordances—the likelihood of a situation eliciting emotional states and behaviors—located in a way that structures the stroll as a mosaic of intermittent motion and stillness giving rise to dynamics of movements that can be experienced as pleasurable and joyful: paths to walk on, benches to sit on, plants unusually close to the sense-maker which encircle a pool in the garden, for example. Accordingly, emotions emerge as specific forms of bodily directedness toward the valences and affordances of a given situation in the engagement with the environment that has affect-like properties: e.g., the emotion of joy is extended over the affordances in the environment, over the sense-maker, her feeling and perceiving body and the joyful situation as a whole. Against this background, it is more accurate to say that the (human or artificial) sense-maker's mode of being in interaction is joyful, instead of merely saying that the sense-maker is joyful.

This contribution reflects upon the conditions for movement-based affective interactions between artificial agents, which are programmed to move, and human agents, which are moved to move, from an embodied and distributed perspective, and answers the question of how a shift of attention from emotion categorization and recognition to the role of movement can contribute to research on affective interactions between human and artificial agents.

## Engaging in Embodied Affectivity with a Human Partner

Affective computing, the discipline that deals with emotions and artificial agents, usually focuses on implementing emotions and giving artificial agents the ability to recognize and express them or to infer the emotional states of a human. Movement information such as speed or acceleration, body posture, and facial expressions are intuitive ways to infer the affective state of a person and are used to extract information about emotions and collect data to classify them, a task which is a challenge to develop emotion-aware robots. Emotions can be classified from body motion patterns in a successful way. Body poses and movements are excellent to convey emotional cues, as Spezialetti et al. (2020) remark. A shift of attention from emotion categorization to the role of movement in the affective engagement of an artificial agent with a human agent focuses on the observation that emotions are embodied motivators, they animate agents to behave because these feel moved to move (de Rivera, 1977). Agents are moved to move toward or against or away from an object, a situation, a partner in interactions. The constitutive role of body motion in the feel of emotion has to be searched in the ongoing interaction with the environment whose qualities co-constitute the experienced feel and in which movement is instrumental to moving the perceiving body toward or out of an emotion-laden situation, changing or influencing the conditions for affective interactions (Scarinzi, 2014). In other words, emotion is not kicking, embracing, or running away. Rather, it is the motivational-affective source of such actions. Fuchs and Koch (2014) label this action readiness as “e-motion”: in an affective interaction; we are moved by movement and moved to move. Being affected by affective affordances of the environment or of a situation triggers a specific bodily resonance (“affection”) which in turn influences the emotional perception and evaluation of the situation and implies a corresponding action readiness (“e-motion”). Despite the motivational–affective explanation of such an approach, it is not based on a linear causality model. The emotion-laden situation is not explained in terms of cause and effect. So, for example, it is not explained in terms of the belief-desire concept like we believe the lion to be dangerous, want to run away and this is our fear of him. Such an explanation is considered to be unable to capture the phenomenal character of emotion. It fails to account for the changing intensity of emotions within the same situation: it seems virtually impossible to indicate what more intense anger, shame, or fear should be without referring to bodily experience (Fuchs and Koch, 2014). For this reason, models of circular causality are considered to be more suitable. Accordingly, emotions result from the body's own feedback and the circular interaction between affective affordances in the environment and the subject's bodily resonance (sensations, postures, expressive movements, movement tendencies). In an effective interaction, our body is tacitly affected by the other's expression, and we experience the kinetics and intensity of our emotions through our own bodily kinaesthesia and sensation. This means that in every affective social encounter, two cycles of embodied affectivity continuously modify each partner's affective affordances and resonance (Fuchs and Koch, 2014).

Against this background, in an affective interaction between a human and an artificial agent, artificial agents do need to be able to take part in two cycles of embodied affectivity by both recognizing how her own body's emotional resonance reacts and how emotion is distributed over body and environment, over the situation as a whole in order to be able to co-determine a situated affective interaction with the human sense-maker while making sense of the affective affordances through her own body. In other words, the artificial agent needs to some degree the similar form and sensorimotor capacities like living bodies and sensations like a lived body. *Organismoid embodiment* (Ziemke, 2001) refers to such types of artificial agents. An artificial agent characterized by organismoid embodiment would be able to take part in an affective interaction because it could be programmed to move and be moved emotionally in an autonomous way. The body would function as a medium of emotional perception not with the purpose of better understanding the human, but with the purpose of co-determining an affective interaction—a cycle of embodied affectivity—and providing the interaction with the condition for feeling how good, how sad, how joyful or how bad the mode of being in the interaction can be in the social encounter. If emotion is grounded in bodily directedness toward the valences and affordances of a given situation, then a robot without the mastery of the two cycles of embodied affectivity could hardly be expected to be able to affective interactions and co-determine an emotion-laden mode of being in the interaction.

As Cañamero (2019) points out, emotions in artificial agents should have their own temporal dynamics and should interact with one another. In an embodied movement-based approach, this would help the organismoid embodied agent understand the body's own feedback to engage in an affective loop with a human agent with “internal” and “external” perception activities co-determined in the interaction itself. As affectively autonomous artificial agents with their own motivation, they would be moved by a movement to move as a consequence of their specific bodily resonance (“affection”) corresponding to action readiness (“e-motion) in affective interactions. For a movement-based affective interaction between artificial and human agents, the environment with its affordances is part of the affective cognitive-emotional system for action readiness of both the artificial agent and the human agent and should be readable by both partners so that they can modify each other's affective affordances and resonance in interaction.

## Conclusion

A shift of attention from emotion categorization and recognition to the role of movement in studying affective interactions between human and artificial agents in the environment means to focus on the constitutive role of body motion in a cycle of embodied affectivity and on how the kinetics and intensity of the emotions of one partner affects the body motion of the other partner and influences her action readiness. In the interaction loop, the partners co-determine and modify the affective affordances of the interaction itself. In a movement–based approach to affective interactions between a human and an artificial agent, the artificial agent is expected to engage in a cycle of embodied affectivity and to contribute to the dynamics of the interaction with

- a specific bodily resonance (the artificial partner's “affection”) in order to be able to influence the way the human partner evaluates the situation and in order to allow or trigger the required corresponding action readiness (the human partner's “e-motion”);
- action readiness (the artificial partner's “e-motion”) corresponding to the emotional perception and evaluation of the situation in order to be able to trigger the corresponding bodily resonance (the human partner's “affection”) in the human partner for allowing the necessary evaluation of the situation.

E-motion and affection become conditions for being programmed to move and to move to emotion in a shared space of co-determined affective affordances.

## Author Contributions

AS contributed to the conception, to developing the main thesis and to structuring the argumentation and wrote the text. LC contributed in a relevant way in interpreting relevant research on emotion and artificial intelligence. Both authors contributed to the article and approved the submitted version.

## Funding

AS was supported by the Fellowship Programme Paris Seine Excellence Initiative IAS FIR (EURAXESS) at the CY Advanced Studies, CY Cergy Paris Université (France) in 2020 and 2021.

## Conflict of Interest

The authors declare that the research was conducted in the absence of any commercial or financial relationships that could be construed as a potential conflict of interest.

## Publisher's Note

All claims expressed in this article are solely those of the authors and do not necessarily represent those of their affiliated organizations, or those of the publisher, the editors and the reviewers. Any product that may be evaluated in this article, or claim that may be made by its manufacturer, is not guaranteed or endorsed by the publisher.
